# Elucidating the mechanisms underlying GATA-1 activity

**DOI:** 10.1186/1756-8935-6-S1-P49

**Published:** 2013-03-18

**Authors:** Roland Gamsjaeger, Ana Silva, Nicholas Shepherd, Lorna Wilkinson-White, Ernest Laue, Gerd Blobel, Jacgui Matthews, Joel Mackav

**Affiliations:** 1 School of Molecular Bioscience, University of Sydney, NSW, Australia; 2 Department of Biochemistry, University of Cambridge, UK; 3 Children’s Hospital of Philadelphia, PA, USA

## 

The fact that GATA-1 binds to gene regulatory elements that contain GATA sites, and thereby ultimately regulates the expression of hundreds of genes involved in hematopoietic development is well established. However, at a molecular level, the steps between DNA binding and changes in the expression levels of target genes are only partly understood, and the goal of our work is understand at a mechanistic level the changes in chromatin structure and occupancy and the protein interactions that are made to drive these changes in gene expression. We have recently shown that, surprisingly, the localization of GATA1 to its target sites in chromatin is dependent on acetylation of specific lysines in the protein, and have elucidated the structural basis for this requirement [1,2]. Our data point towards a model for gene regulation in which not only are the post-translational modifications of histones essential for establishing gene expression patterns, but equivalent modifications in DNA-binding transcription factors are also an integral part of whatever ‘code’ underlies these patterns.

Concurrently, we have also determined the molecular details underlying the recruitment of the Nucleosome Remodeling and Deacetylase (NuRD) complex to GATA1-dependent gene promoters [3], an event that is essential for normal GATA1 activity. Combined with data that explore the molecular makeup of the NuRD complex, these results provide a glimpse into the mechanisms through which complex coregulator complexes are recruited to target genes and begin to map out the molecular events that drive gene regulation both in erythropoiesis and, by extension, in a range of other tissues.
